# Impact of High Seas Closure on Food Security in Low Income Fish Dependent Countries

**DOI:** 10.1371/journal.pone.0168529

**Published:** 2016-12-29

**Authors:** Louise S. L. Teh, Vicky W. Y. Lam, William W. L. Cheung, Dana Miller, Lydia C. L. Teh, U. Rashid Sumaila

**Affiliations:** 1 Fisheries Economics Research Unit, Institute for the Oceans and Fisheries, The University of British Columbia, Vancouver, Canada; 2 Changing Ocean Research Unit & Nereus project, Institute for the Oceans and Fisheries, The University of British Columbia, Vancouver, Canada; University of Hong Kong, HONG KONG

## Abstract

We investigate how high seas closure will affect the availability of commonly consumed food fish in 46 fish reliant, and/or low income countries. Domestic consumption of straddling fish species (fish that would be affected by high seas closure) occurred in 54% of the assessed countries. The majority (70%) of countries were projected to experience net catch gains following high seas closure. However, countries with projected catch gains and that also consumed the straddling fish species domestically made up only 37% of the assessed countries. In contrast, much fewer countries (25%) were projected to incur net losses from high seas closure, and of these, straddling species were used domestically in less than half (45%) of the countries. Our findings suggest that, given the current consumption patterns of straddling species, high seas closure may only directly benefit the supply of domestically consumed food fish in a small number of fish reliant and/or low income countries. In particular, it may not have a substantial impact on improving domestic fish supply in countries with the greatest need for improved access to affordable fish, as only one third of this group used straddling fish species domestically. Also, food security in countries with projected net catch gains but where straddling fish species are not consumed domestically may still benefit indirectly via economic activities arising from the increased availability of non-domestically consumed straddling fish species following high seas closure. Consequently, this study suggests that high seas closure can potentially improve marine resource sustainability as well as contribute to human well-being in some of the poorest and most fish dependent countries worldwide. However, caution is required because high seas closure may also negatively affect fish availability in countries that are already impoverished and fish insecure.

## Introduction

Food security, as defined at the 1996 World Food Summit, exists when “all people, at all times, have physical and economic access to sufficient, safe and nutritious food that meets their dietary needs and food preferences for an active and healthy life”. Feeding the world’s expected population of 9 billion people by 2050 is a pressing global issue [1]. Despite progress in reducing hunger over the past decade, around 795 million people remain undernourished in 2015, with the situation being more pronounced in Southern Asia and Sub-Saharan Africa [2,3]. Against this backdrop, several authors have recently stressed the need to consider the role fish can play in contributing to securing food and nutritional security for the world’s growing population [1,4].

Currently, fisheries and aquaculture provide up to 3 billion people with almost 20% of their average per capita animal protein intake [5]. Due to the affordability of fish relative to other protein sources, it is especially crucial for the food and nutritional security of coastal communities in poor, low development countries. In fact, almost three-quarters of countries where fish is an important source of protein (defined here as contributing to more than one-third of total animal protein supply) are low-income, food deficient countries [6]. Moreover, fish is also part of the staple diet for people in some developed countries. Yet, the food security for some of the world’s poorest populations is threatened by the current degraded state of global fisheries, and the situation is expected to be amplified by the impacts of future climate and socio-economic change [7].

The sustainability of high seas fisheries is of concern because of increasing fishing pressure, inadequate management, and the tendency for deep sea fishes to have long lived life histories which make them vulnerable to overfishing [8,9]. Some high seas species, especially commercially important tunas and billfishes, forage both in the high seas and Exclusive Economic Zones (EEZs) of coastal nations. Overexploitation of high seas fish stocks can therefore affect the availability of fish in countries’ EEZs. Recent proposals to close the high seas to fishing have indicated that this may be beneficial for the rebuilding of fish biomass, increase the quantity and improve the distributional equality in global fisheries catch, and increase the resilience of fish stocks to climate change [10–12]. For instance, Sumaila et al. [11] found that biomass spillover from closing the high seas would benefit the domestic fisheries in 120 maritime countries under a scenario in which post high seas closure catches increased by 42%. At the same time, it would result in net losses for 65 countries, particularly those which specialise in fishing the high seas, such as Japan, China, and Spain.

Although prior research has identified winners and losers from closing the high seas, how this closure will impact food security for the poorest and most fish dependent countries is not clear. As such, this paper aims to answer the research question: How will high seas closure affect the availability of domestically consumed fish in fish reliant, low income countries? Our approach is to first identify which countries will be positively and negatively affected by high seas closure. Then, we assess whether the effect of high seas closure will impact upon locally consumed food fish. While there are four dimensions to food security–food availability, economic and physical access to food, stability over time, and food utilization [13], we focus on the availability aspect of food security in this study.

## Methods

### Projected changes in catch of straddling fish taxa due to high seas closure

Spillover of fish biomass from high seas closure is expected to affect the catch of straddling fish taxa (i.e., fish species which straddle the border between each country’s Exclusive Economic Zone (EEZ) and the high seas) in coastal countries [11]. In a previous study, Sumaila et al. [11] projected changes in global fisheries catch under five scenarios of increase in straddling taxa catch within EEZs (10%, 18%, 20%, 42%, and 70% increases) following high seas closure to fishing. In this study, we leave out the extreme scenarios (10% and 70%), which were used for sensitivity analysis, and choose to focus on the mid-range scenarios of 20% and 42% projected increase in straddling taxa catch (we leave out 18% due to its proximity to 20%).

The results from Sumaila et al. [11] did not identify the fish taxa or groups associated with predicted changes in catch at the country level. High seas closure is expected to positively affect the biomass of straddling fish stocks. Therefore, we assume that positive changes in catch resulting from high seas closure relate to the catch of straddling stocks. A list of straddling fish taxa caught by coastal countries globally was provided by [14], which is used as the basis for this analysis.

### Fish Dependency

The majority of countries which are highly dependent on fish for protein are low-income, fish deficient countries [6]; therefore, this study focuses on two groups of countries: 1) countries that are highly fish dependent; and 2) low-income, least developed countries (LDCs). Data for determining the fish dependency of countries was obtained from [6] and the Food and Agriculture Organization of the United Nations (FAO) Statistics Division (FAOSTAT, http://faostat3.fao.org), which provides data on the quantity of animal protein supply (g/capita/day) from different sources, including fish, seafood, and meat. Following [6], we calculated fish dependency as the percentage of fish and seafood out of total animal protein supply. Countries with fish dependency of more than 30% were identified as high fish dependent countries (HFDCs). The United Nations categorises 43 nations, included those that are land-locked, as ‘least developed”. In this study we only include the 32 maritime countries for which predicted catch and landed values from high seas closure were available from the study by [11].

### Benefits from high seas closure

We defined two types of benefits arising from high seas closure. First, countries could benefit directly through an increase in the supply of fish to the local population. This would occur if projected catch gains following high seas closure consisted of the same type of fish that are commonly consumed by local populations. Second, indirect food security benefits could still arise if the projected catch gains involved species that are not consumed domestically but are used for trade or other purposes; these economic activities would, in principle, contribute to the revenues of citizens and national governments, which would allow for improved economic opportunities for local populations, thereby providing them with income necessary for purchasing food. Importantly, we assume that fisheries within each EEZ are managed well, thereby enabling the benefits from high seas closure to be realised.

#### Direct benefit—Domestic consumption of straddling fish taxa

We assessed whether projected changes in fish catch following a high seas closure involved the same type of fish that are consumed by the local population, or whether changes consisted of fish species that are primarily exported or targeted by foreign fishing vessels fishing within the country’s EEZ. To determine this, catches of straddling fish taxa from each of the assessed countries were extracted from the *Sea Around Us* catch database (www.seaaroundus.org) for 2006, which was the most recent year for which data was available at the time of the analysis by [14]. We then reviewed the literature, both primary and grey, to identify the main uses of straddling fish taxa in each country. High seas closure was determined to have a direct impact on domestic food supply, and hence food security, if the straddling species was a fish that was commonly consumed by the local population. Likewise, the direct impact of high seas closure was assumed to be minimal if the straddling species was predominantly used for export, or caught by foreign fishing fleets.

By fish supply, we refer to the country’s annual fisheries catch. We acknowledge that the nature of each country’s fish marketing chain will affect the final amount of fish made available to the local population; however, it is beyond the scope of this paper to account for differing market systems. As such, we assumed that for each country, catches of species that are commonly consumed food fish by the local population will mainly be used domestically. Note that this does not assume that the same fish species may not be used for export or other purposes.

An exception was made for the case of tunas in the Pacific Island Countries and Territories (PICTs). Nearshore pelagics make up between 20–30% of the total coastal fishery catches of the 6 PICTs analysed in this study, although tuna dominates the nearshore pelagic catch only in Kiribati [15]. These coastal fisheries take only a tiny fraction of the regional catch of skipjack and yellowfin tuna, the vast majority of which are targeted by industrial fisheries fishing offshore [16,17], and which do not contribute to the domestic fish supply of PICTs [18]. Further, fish and invertebrates from reefs, mangroves, and other nearshore habitats dominate the catch targeted for subsistence [16,19]. Therefore, although consumed domestically, we treat the catches of tunas and other large pelagics in PICTs as industrial fisheries targeted for export, and not for domestic consumption.

#### Indirect benefit–Economic value of projected catch

Countries where straddling taxa are not consumed domestically could still potentially obtain food security benefits indirectly through increases in economic activity and household incomes arising from projected increases in fisheries catch, thereby improving the ability for people to purchase food. To capture this effect, we used economic and income multipliers estimated by [20]. These multipliers reflect the impact a change in fisheries output will have on fisheries related economic activities and the household income of fishery workers, and were estimated for all maritime countries globally. Projected percentage changes in landed value relative to the status quo were taken from [11] for each of the two high seas catch scenarios. We estimated the economic and household income effect associated with projected increases in landed value as follows:

Income effect: LV% x income multiplier;
Economic effect: LV% x economic multiplier;

Where LV% is the projected change in landed value under each catch gain scenario [11], and income and economic multipliers were taken from [20]. We used the calculated income and economic effect as an indicator of the indirect food security benefits arising from high seas closure for countries which did not benefit directly in terms of an increase in domestically consumed fish.

### Mitigating losses from high seas closure

#### Mitigating direct loss in fish catch—Alternative fish (i.e. non-straddling taxa) and non-fish food sources

A concern for countries with projected catch losses arising from high seas closure is whether alternative fish and non-fish food sources are available in the event of decreased supply of straddling fish species. Alternative fish sources include inland, freshwater, or reef fisheries, or aquaculture. Agriculture could also compensate for the shortfall in fish supply, notwithstanding the difference in nutrients obtained. The availability of food safety programmes, such as those operated by food aid agencies or national governments, could also mitigate the fish supply shortfall. Further, high levels of adaptive capacity, which encompasses human capital, governance effectiveness, and social capital, may indicate a better ability to carry out planned adaptation to future shocks and changes [7], such as changes in food supply.

To investigate the potential for countries to mitigate the impact of decreased straddling fish taxa supply, we reviewed the literature to document the presence of mitigating factors and indicators in 21 countries projected to experience net catch losses under the various scenarios. We undertook a qualitative comparison, assuming that a higher presence of the 7 factors listed below represented a better opportunity for the country to cope with the impact of decreased straddling fish taxa supply:

1. Aquaculture;
2. Inland fisheries;
3. Reef and coastal fisheries;
4. Food safety net programmes–this indicator measures the presence of public initiatives provided by non-governmental organisations (NGOs), government or other multilateral agencies to protect the poor from shocks to food supply for the year 2015. It is a qualitative score provided by the Global Food Security Index (foodsecurityindex.eiu.com). 0 = minimal programmes run only by NGOs or multilateral agencies; 1 = moderate presence of programmes run mainly by NGOs or multilateral agencies; 2 = moderate prevalence and depth of programmes run by the government, multilateral agencies, or NGOs; 3 = national coverage with very broad but not deep coverage of programmes run mainly by government with some reliance on NGO or multilateral agency support; 4 = presence of national government run programmes, with minimal support required by NGOs or multilaterals;
5. National level of adaptive capacity obtained from [7];
6. Percentage of agricultural land that is equipped for irrigation for the year 2011– this is an indicator of a country’s exposure to food supply shock [13], and was obtained from FAOSTAT (http://faostat3.fao.org);
7. Livelihood diversification by fishers–we documented whether, in general, fishers in the respective countries also engaged in other food producing activities, such as farming or livestock rearing. Having a diversified livelihood acts as a buffer which enables households to grow or buy food in the event of external environmental or socio-economic shocks.

The presence of alternate food sources may not be able to supplement or make up for decreased straddling fish supply if those food systems are themselves under pressure to fulfil national food security demands. To account for this, we used the Global Food Security Index (GFSI) to gauge a country’s general food security status. The GFSI (www.foodsecurityindex.eiu.com) score for each country incorporates three dimensions of food security: affordability, availability, and quality, and ranged from 0 (low) to 100 (high food security).

#### Indirect mitigating factors—Income equality and governance effectiveness

In addition to obtaining alternative sources of food, coastal communities in countries projected to experience losses in catch may still be able to secure sufficient food if there is a conducive economic environment which enables them to improve their incomes for buying or accessing food (i.e., there is a trickle down effect from national governments to local communities), or national governments provide the appropriate support and investment for enhancing food security [13]. To account for this, we looked at two national level indicators:

1. Gini coefficient: this is an indicator of income equality within a country (0 = perfect equality, 1 = perfect inequality). Income inequality decreases the ability of poor households to stay healthy and to move out of poverty because it hampers their ability to accumulate human and physical capital [21]. As such, we expect that opportunities for coastal communities in a country with a low Gini coefficient may be relatively better in terms of receiving economic and/or food security support and services from national governments compared to a country where the Gini coefficient is high. Global Gini coefficient data represented by a Gini index was obtained from the World Bank World Development Indicators [22].
2. Governance: Good governance is key to food security [5]. The Global Governance Index developed by the World Bank provides a score for six different aspects of governance, including voice of accountability, political stability, government effectiveness, regulatory quality, rule of law, and control of corruption [23]. Data for these indicators were obtained from the World Bank (http://info.worldbank.org/governance/wgi) for the year 2014. Of these, we chose 3 of the most relevant governance aspects that might affect the ability of local communities to obtain the necessary support to improve their food security situation. These included:
  - Government effectiveness–indicates the quality of public services, the quality of civil service and its independence from political interference, quality of policy formulation and implementation, and the credibility of governments’ commitments to such policies [23].
  - Control of corruption–captures the extent to which public power is used for private gain, as well as “capture” of the state by elites and private interests [23].
  - Political stability–captures the likelihood that the government will be destabilised by unconstitutional means [23].

## Results and Discussion

### Fish Dependency

The contribution of fish to total animal protein supply for the 46 countries included in the present analysis is summarised in Table 1. Note that data from [24], [25] and [26] were used for PICTs and African countries for which fish protein contribution was not available from FAO and [6]. The top 10 fish dependent countries are located in South and Southeast Asia, West Africa, or are island nations in the Western Pacific and Indian Oceans. Of the 32 least developed countries (LDCs), 18 were also considered to be high fish dependent countries, and are hereafter referred to as high fish dependent LDCs (HFDLDCs). Among LDCs, Sierra Leone had the highest fish dependency (76%), while others, mainly in Africa, had minimal fish dependency. Despite the low fish consumption rates in some of these countries, national governments are trying to promote fish as an alternative protein source in countries such as Eritrea, thereby reiterating the importance of fish for future food security.

**Table 1:** Contribution (%) of fish to total animal protein supply and domestic use of straddling fish taxa in Least Developed Countries (LDC), High Fish Dependent countries (HFDC), and High Fish Dependent LDCs (HFDLDC). Countries are listed in order of fish dependency.

Country	Fish protein %	LDC	HFDC	HFDLDC	Domestic use of straddling fish taxa
Solomon Islands^1^	92			√	*
Kiribati^1^	84			√	*
Maldives	76		√		√
Sierra Leone	76			√	
Tuvalu^1^	71			√	*
Cambodia	65			√	
Equatorial Guinea^2^	62			√	√
Comoros	57			√	√
Vanuatu^1^	56			√	*
Bangladesh	56			√	√
Indonesia	53		√		√
Gambia	49			√	√
Sao Tome Principe	48			√	
Seychelles	48		√		
Sri Lanka	44		√		√
Senegal	44			√	
Japan	43		√		√
Togo	43			√	
Philippines	43		√		√
Myanmar	42			√	
Korea Rep	38		√		√
Thailand	38		√		√
Malaysia	37		√		√
Mozambique	37			√	
Cameroon	36		√		√
Vietnam	34		√		
Cote d'Ivoire	35		√		√
Benin	35			√	√
Nigeria	35		√		
Guinea	33			√	√
Fiji	32		√		*
Congo Dem Rep^2^	31			√	√
Samoa	25	√			*
Angola	25	√			√
Tanzania	24	√			√
Madagascar	16	√			√
Haiti	12	√			
Mauritania	10	√			√
Yemen	7	√			√
Timor Leste	7	√			
Liberia	6	√			√
Djibouti	4	√			√
Guinea Bissau	4	√			
Somalia	3	√			√
Sudan	<1	√			
Eritrea^3^	<1	√			√

^1^ Source: [24].

^2^ Source: [25].

^3^ No data in FAOSTAT. Fish plays a very minor role in the national diet (citation removed). Therefore we assigned Eritrea a fish protein % that was equal to the lowest percentage of all assessed countries (<1% for Sudan).

* Straddling taxa (i.e., tunas) are not treated as being used for domestic consumption because a much larger quantity of tunas is taken by industrial fleets, relative to local coastal fisheries. The industrial catch does not contribute to local fish supply in the PICTs.

### Direct food security benefit from high seas closure—Domestic use of straddling fish taxa

#### Breakdown by country groups

**Fig 1:**
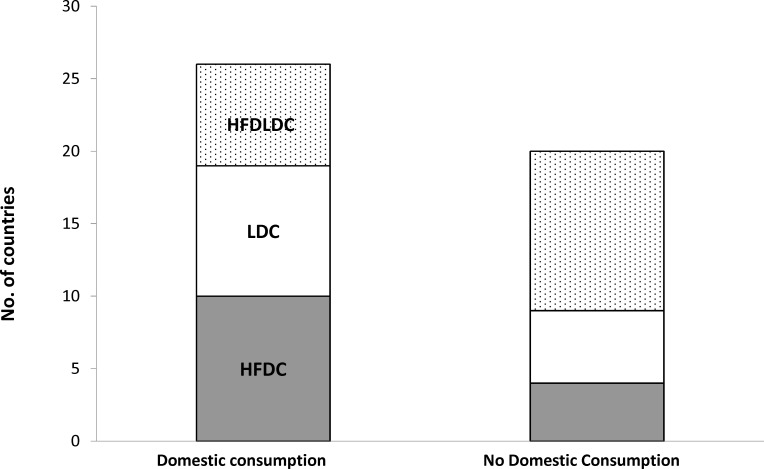
Total number of countries that use straddling species for domestic consumption, broken down according to least developed (LDC), high fish dependent (HFDC), and high fish dependent LDCs (HFDLDC).

Twenty-six (56%) of the assessed countries used straddling species for domestic consumption (Fig 1). Seventy-one percent of HFDCs made use of straddling species locally, compared to 39% of highly fish dependent LDCs (HFDLDCs) and 64% of LDCs. This indicates that high seas closure may have the largest effect on domestic fish supply in HFDCs.

#### Projected net catch gains and losses

Seventy percent of assessed countries were projected to experience net catch gains under both scenarios, with average increases ranging from 13–31% relative to the status quo. Of these countries, slightly above half (56%) used straddling taxa locally. Another 24% of assessed countries had projected losses under both scenarios, with average decreases ranging from -53 to -47% relative to the status quo. Fourty-five percent of these countries used straddling taxa locally. Therefore, high seas closure was projected to have a relatively more positive than negative impact on low income fish dependent countries because the number of countries projected to gain from high seas closure was larger than those projected to lose. However, it is noted that the magnitude of projected losses in catch exceeded the projected gains. In addition, more than half the countries projected to experience losses were classified as least developed countries, making the projected negative impact particularly damaging to the already impoverished state of these countries.

Across both scenarios, 55–60% of those countries projected to gain would potentially see a benefit in terms of local fish availability (Table 2). When considered among all 46 assessed countries, about 40% (39–46%) of the countries would potentially benefit from increased local fish availability. Among the countries projected to lose from high seas closure, the proportion that relied on straddling taxa domestically ranged from 45% to 57%. As a percentage of all assessed countries, 11% to 17% of countries, depending on scenario, would potentially see local fish availability decline due to high seas closure.

**Table 2:** Number of countries with projected gains and losses under each high seas catch scenario, and the corresponding number of countries which consume straddling fish taxa domestically (denoted by No. domestic use).

	Scenario (% increase in straddling taxa catch)			
	20%		42%	
	Gain	Loss	Gain	Loss
No. countries	32	14	35	11
No. domestic use	18	8	21	5
% Domestic use/total assessed countries	39	17	46	11

**Fig 2:**
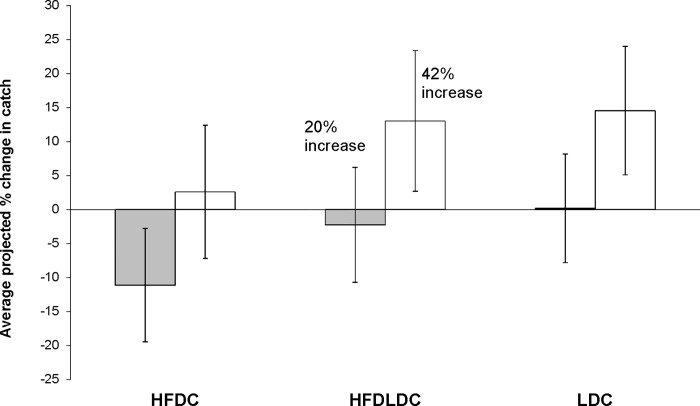
Average projected % change in catch (±standard error) for Least Developed Countries (LDCs), High Fish Dependent Countries (HFDCs), and High Fish Dependent LDCs (HFDLDCs) under 2 scenarios of catch gains following high seas closure.

An increase of at least 18% in catch of straddling taxa following a high seas closure was expected to result in net gains in global catch relative to the status quo [11]. However, when considering all fish dependent and low income countries as a group, we find that on average, these countries would collectively experience net gains in catch (relative to the status quo) only under the scenario of 42% catch gain following high seas closure (Fig 2).

On average, countries where straddling taxa were not consumed locally were projected to experience a net loss of -6% and gain of 11% in catch relative to the status quo under the 20% and 42% scenarios, respectively. This was fairly similar to countries which used straddling taxa locally, for which projected changes in catch were -3% and 10% relative to the status quo under the 20% and 42% scenarios, respectively (S1 Table).

Under the 42% catch increase scenario, projected changes in catch relative to the status quo were not high. Among countries which used straddling taxa domestically, average net catch gains of 14% were projected for LDCs, while similarly minimal gains of 8% and 7% was projected for HFDCs and highly fish dependent LDCs, respectively. Among countries that did not consume straddling taxa domestically, HFDLDCs and LDCs were projected to experience average net gains of 18% and 14%, respectively, whereas HFDCs were projected to experience an average net loss of 11%. While countries that do not consume straddling taxa domestically may not directly benefit from high seas closure, the projected changes in catch may still indirectly affect food security via the economic impact on local communities through the increase in secondary and tertiary activities and services, e.g., processing [20].

#### Least Developed Countries (LDCs)

About sixty percent (64%) of LDCs made use of straddling taxa locally. The majority (79%) of LDCs were projected to experience net gains across both 20% and 42% catch gain scenarios, with average increases of 15% and 32% relative to the status quo, respectively. Of these countries, 80% used straddling taxa domestically. Samoa, Tanzania, and Yemen were the three LDCS with the highest projected losses across both scenarios, with an average of -54% and -49% loss relative to the status quo under the 20% and 42% scenarios, respectively. Although projected losses are very high for Samoa (around -90% relative to the status quo under both scenarios), high seas closure would likely not substantially affect the supply of domestically consumed fish because the main straddling species caught is tuna, which is primarily caught offshore by longliners and exported [27]. In contrast, high seas closure may affect the supply of locally consumed food fish in Tanzania and Yemen (S2 Table), where straddling taxa such as Indian mackerel, Spanish mackerel, and tunas are commonly preferred species [28,29].

#### High Fish Dependent LDCs

Among the 18 HFDLDCs, 7 (39%) used straddling taxa domestically. Eleven countries were projected to experience net catch gains across both scenarios, with average increases of 17% and 36% under the 20% and 42% catch gain scenarios, respectively. Five of these countries, mainly located in Africa, consumed straddling fish taxa locally (Bangladesh, Congo Democratic Republic, Gambia, Guinea, and Equatorial Guinea). This is a positive sign in terms of fish protein security, given that per capita fish availability has been decreasing in much of sub-Sahara Africa [30]. The domestically consumed fish species in these countries were mainly small, low-value species such as herring, sardinella, and Hilsa shad (Bangladesh) that are affordable for poor rural coastal communities. For this group of countries, high seas closure could likely increase the availability of important food fish for local populations. This is also particularly important for supporting the nutritional requirements of poor populations, as small fish have high nutrient content, e.g., Omega-3, vitamin A, iron, zinc, and calcium, which can potentially reduce micronutrient and essential fatty acid deficiencies among the undernourished [30].

Four HFDLDCs (Comoros, Togo, Kiribati, and Vanuatu) were projected to incur catch losses across both 20% and 42% scenarios, with average decreases of -43% to -39% relative to the status quo, respectively. Straddling taxa are not consumed locally in Togo and Vanuatu; consequently, high seas closure may only affect the availability of domestically consumed fish in Comoros and Kiribati. While sardinella, which made up about 20% of Comoros’ total straddling taxa catch, is consumed domestically, skipjack tuna, which made up 57% of straddling taxa catch, is mainly caught by foreign fishing fleets and not landed in Comoros [31]. Moreover, even though tunas are caught by local artisanal fishers [32], many coastal fishing communities in Comoros prefer the taste of reef fishes and believe them to be superior to pelagic species in terms of nutritious value [33].These factors may therefore dampen the projected impact of high seas closure on local Comoros fish supply.

Two negatively affected HFDLDCs (Kiribati and Vanuatu) are Pacific island states where the main straddling species are tuna that are primarily caught by foreign fleets or for export. Although nearshore pelagics make up around 21% of total coastal catches in Kiribati and Vanuatu [15], small-scale fishing for tuna occurs only in Kiribati, but not Vanuatu [18]. The total quantity of tunas caught by the foreign fleet dominated industrial fisheries in Kiribati and Vanuatu far exceed the amount taken by local nearshore fisheries [17,34]. Thus, closing the high seas may have a proportionately larger effect on tunas caught by foreign fleets within the EEZs of these 2 PICTs relative to the amount caught for domestic consumption. Further, demersal reef fish, the bulk of which are caught for subsistence, make up the majority of coastal fisheries catches in Pacific island states [15]. As such, high seas closure may have a minimal direct effect on local food security in highly fish dependent LDCs where catches are projected to fare the worst. However, there may be indirect impacts on food security because access fees paid by foreign fishing vessels to fish within the EEZs of these countries contribute substantially to national revenues. For instance, fishing access fees totalling USD 47.4 million made up approximately half of Kiribati’s total government revenue in 2012 [35], and about 25% of its gross domestic product [17]. Further, increasing the use of offshore tuna stocks to supply local markets in Pacific islands was identified as a means of adapting to potential climate change impacts on coral reef fisheries [16,24]. Therefore, the projected loss in tuna catches still poses an indirect food security concern for the PICTs.

For the remaining highly fish dependent LDCs with projected catch gains, the main straddling species also consisted of tuna and other large pelagics that were primarily exported or caught by foreign vessels (Table 3). In Cambodia, which is among the countries with highest projected catch gains and fish dependency, high seas closure may nevertheless not have any large noticeable effect on local fish supply as the majority of fish consumed in the country is from inland fisheries [36]. Thus, overall projected increases in catch of straddling taxa from high seas closure may not substantially increase local fish supply for highly fish dependent LDCs, which are likely the countries with the most urgent need for an increased supply of fish as an affordable protein source.

**Table 3:** Summary of main straddling fish taxa caught by each country, and whether the fish taxa are consumed domestically.

Country	Country Group	Main straddling taxa^1^	Domestically consumed?	Source (s)
Angola*	LDC	Sardinella, Cunene horse mackerel, Chub mackerel	Yes	[37]
Bangladesh*	HFDLDC	Hilsa shad	Yes	[38,39]
Benin†	HFDLDC	Little tunny, Swordfish	Yes	[40]
Cambodia*	HFDLDC	Marine crabs, Cephalopods	No	[36]
Cameroon*	HFDC	Sardinella, Largehead hairtail, Barracudas	Yes	[41]
Comoros†	HFDLDC	Skipjack tuna, Sardinella	Yes	[31]
Congo Dem Republic*	HFDLDC	Sardinella	Yes	[42]
Cote d'Ivoire*	HFDC	Skipjack tuna	Yes	[43]
Djibouti*	LDC	Jacks and pompanos, Barracuda, Seerfishes	Yes	[44]
Equatorial Guinea*	HFDLDC	Herrings	Yes	[45]
Eritrea*	LDC	Barracudas, Sardinellas, Jacks and pompanos, Indian mackerel, Queenfishes, Requiem sharks	Yes	[26,46]
Fiji†	HFDC	Albacore, Yellowfin tuna	Yes^2^	[47,48]
Gambia*	HFDLDC	Sardinella	Yes	[49]
Guinea Bissau*	LDC	Jacks and pompanos, West African Spanish mackerel, Marine crabs	No	[50]
Guinea*	HFDLDC	Sardinella, Jacks	Yes	[51]
Haiti*	LDC	Marine crabs	Yes	[52]
Indonesia*	HFDC	Skipjack tuna, Goldstripe sardinella, Narrow-barred Spanish mackerel	Yes	[53]
Japan†	HFDC	Chub mackerel, Japanese anchovy, Skipjack tuna	Yes	[54]
Kiribati†	HFDLDC	Skipjack tuna, Jacks and pompanos	Yes^2^	[55] [15]
Korea†	HFDC	Skipjack tuna, Flying squid	Yes	[56,57]
Liberia*	LDC	Sardinella, Barracudas, Blue butterfish	Yes	[58]
Madagascar*	LDC	Narrow-barred Spanish mackerel, Marine crabs	Yes	[59,60]
Malaysia†	HFDC	Indian scad, Kawakawa, Torpedo scad, Jacks and pompanos	Yes	[61]
Maldives*	HFDC	Skipjack tuna	Yes	[62,63]
Mauritania*	LDC	European anchovy, Sardinella, European pilchard, Octopuses	Yes	[45,64]
Mozambique*	HFDLDC	Yellowfin tuna, skipjack tuna	No	[65,66]
Myanmar*	HFDLDC	No straddling taxa	n/a	[67]
Nigeria*	HFDC	Swordfish	No	[68]
Philippines*	HFDC	Sardinella, Frigate tuna, Skipjack tuna	Yes	[69]
Samoa†	LDC	Albacore, Yellowfin tuna, Bigeye tuna	Yes^2^	[27,70]
Sao Tome Principe*	HFDLDC	Atlantic sailfish, Little tunny, Swordfish	No	[71,72]
Senegal*	HFDLDC	Skipjack tuna, Bigeye tuna	No	[45,73]
Seychelles†	HFDC	Skipjack tuna, Bigeye tuna	No	[74]
Sierra Leone*	HFDLDC	Albacore, Bigeye tuna	No	[75]
Solomon Islands*	HFDLDC	Skipjack and yellowfin tuna	Yes^2^	[76]
Somalia*	LDC	Cephalopods	Yes	[77]
Sri Lanka†	HFDC	Skipjack tuna, Trevally	Yes	[78,79]
Sudan*	LDC	Spanish mackerel	No	[80]
Tanzania†	LDC	Indian mackerel, Sardinella, Yellowfin tuna, Jacks and pompanos	Yes	[28,81]
Thailand*	HFDC	Anchovies, Sardinella, Indian scad	Yes	[82]
Timor Leste*	LDC	Yellowfin tuna	No	[83]
Togo†	HFDLDC	Bigeye tuna	No	[84,85]
Tuvalu*	HFDLDC	Skipjack and yellowfin tuna	Yes^2^	[86,87]
Vanuatu†	HFDLDC	Skipjack tuna, Albacore	No	[88]
Vietnam*	HFDC	Cephalopods, marine crabs	No	[89]
Yemen†	LDC	Yellowfin tuna, Barracudas, Jacks and pompanos, Indian mackerel, Spanish mackerel, Indian oil sardine	Yes	[29,90]

^1^ Source: [14].

^2^ Tunas are consumed domestically in these PICTs, but the bulk of tuna catches in the EEZs are taken by industrial fisheries, which do not contribute to local food security. Consequently, straddling fish taxa are not considered to be used domestically.

* and † indicate countries with projected catch gains and losses, respectively, across both scenarios of increase in straddling taxa catch.

#### High Fish Dependent Countries (HFDCs)

The majority (71%) of HFDCs made use of straddling taxa domestically. These countries were mainly located in Asia and Africa, with the remainder located in the Indian and Pacific Oceans (Table 3). With the exception of Japan and Korea, the other HFDCs are developing countries which generally have large populations of rural and poor fishing communities who rely on fish as the major source of food and livelihood. In particular, countries in Southeast Asia, especially the Philippines and Indonesia, and countries of western Africa, have the highest nutritional dependence on fish and marine ecosystems [91]. As such, the catch of straddling taxa is paramount to supporting the economic as well as social well-being in coastal areas of these countries.

Eight HFDCs (Cameroon, Cote d’Ivoire, Indonesia, Maldives, Nigeria, Philippines, Thailand, and Vietnam) were projected to experience net gains in catch across both scenarios, with average projected catch increases of 9% and 26% relative to the status quo under the 20% and 42% scenarios, respectively (S3 Table). Among this group, straddling taxa were consumed domestically in 6 countries, with small pelagics such as sardinellas and scads and skipjack tuna being the most common straddling species consumed (Table 3).

Another 4 HFDCs were projected to experience catch losses across both 20% and 42% catch gain scenarios (Fiji, Seychelles, Sri Lanka, and Korea), with losses of -53% to -45% relative to the status quo, respectively (S3 Table). Of these countries, straddling taxa were consumed locally in Sri Lanka, Fiji, and Korea. Fiji and Sri Lanka were the HFDCs with highest projected catch losses, averaging 51% and 43% across both scenarios, respectively. In Fiji, albacore and yellowfin tunas are the main straddling species, and albacore is also commonly consumed either fresh or canned among the local population [48]. While high seas closure may affect the availability of this fish source, the impact on overall fish availability may be minimal because Fijian coastal communities rely heavily on reef fisheries and gleaning for subsistence and artisanal purposes [92]. Similarly, both types of straddling species in Sri Lanka–skipjack tuna and trevally, are consumed locally, accounting for 10% and 5.5% of monthly household fish consumption, respectively. Therefore, high seas closure may decrease the supply of fish to local communities, although not by a large extent.

Catch was projected to decrease by around 34% for Korea, where local consumption of seafood, including tuna and squid, is high. The negative impact of high seas closure may be offset to a certain degree in Korea due to its large distant water fleet, as catches from Korea’s distant water fleet are generally consumed in Korea [57]. However, this depends on how the fishing grounds of Korea’s distant water fleet will be affected by high seas closure. On the whole, projected decreases may not have a heavy negative impact on this group of countries.

The most negatively affected HFDC with the highest projected losses but limited domestic straddling taxa dependence was Seychelles, where the dominant straddling taxa–skipjack tuna–is primarily caught by foreign fishing fleets and processed for export. Coastal communities in the Seychelles generally fish on coastal reefs for demersal fish, invertebrates, and nearshore pelagics for subsistence and to supply local markets [93]. Thus, the negative impact of high seas closure may not have a large effect on local fish supply, although the projected decrease in tuna catches may have reverberating economic effects on local communities since the Indian Ocean Tuna canning factory is the country’s largest single employer [74].

### Indirect food security benefits of high seas closure

Fourteen countries with projected catch gains did not consume straddling taxa domestically, but could potentially improve their food security indirectly through the projected increase in revenues, incomes and profits generated by straddling taxa. Half of these countries were located in Africa, with the remainder being Asian, Pacific island, or Caribbean countries (Table 4). Projected landed value gains for these countries ranged from a low of 2.4% to 24% relative to the status quo, under the 20% catch gain scenario, and from 10% to 51% under the 42% catch gain scenario (Table 4). More than half (57%) of the countries were highly fish dependent LDCs (HFDLDCs), and another 29% were LDCs. On the other hand, 42% of the countries with projected losses did not consume straddling taxa domestically. Most of these countries were Pacific Island states, and would stand to suffer indirect food security losses, through the loss in trade of straddling taxa or reductions in fishing access fees.

**Table 4:** Estimated economic and household income effects arising from projected gains in landed value.

Country	Projected % gain in Landed Value^1^		Income multiplier^2^	Economic multiplier^2^	Income effect(%LV x multiplier)		Economic effect(% LV x multiplier)	
	20% scenario	42% scenario			20% scenario	42% scenario	20% scenario	42% scenario
Sierra Leone	2.38	9.94	0.32	0.32	0.76	3.16	0.76	3.16
Mozambique	7.26	27.70	0.74	1.83	5.41	20.64	13.31	50.77
Senegal	7.28	16.72	0.84	2.21	6.13	14.07	16.09	36.95
Sudan	10.52	22.30	0.72	2.95	7.55	15.99	31.05	65.80
Timor Leste	10.86	23.02	0.59	2.11	6.44	13.64	22.94	48.60
Nigeria	11.36	24.34	0.05	0.28	0.62	1.34	3.22	6.91
Guinea Bissau	12.62	26.80	0.32	1.52	4.00	8.49	19.22	40.81
Sao Tome and Principe	22.23	47.10	0.77	2.96	17.02	36.06	65.88	139.59
Myanmar	24.05	50.96	0.32	0.85	7.81	16.55	20.42	43.28
Haiti	24.05	50.96	0.28	1.22	6.85	14.51	29.22	61.93
Cambodia	24.05	50.96	0.54	1.73	12.94	27.43	41.69	88.33
Vietnam	24.05	50.96	0.77	3.47	18.45	39.09	83.38	176.69
Solomon Is.	24.05	50.96	0.65	3.34	15.57	32.98	80.42	170.40
Tuvalu	24.05	50.96	0.65	3.34	15.57	32.99	80.42	170.40

^1^ Source: [11]

^2^ Source: [20]

The economic and income multipliers indicate the impact an increase in fisheries output will have on fisheries related economic activities and the household income of fishery workers [20]. Income multipliers for all countries ranged from 0.05 to 0.84, while economic multipliers ranged from 0.28 to 3.34. This means that, depending on the country, a one dollar increase in fisheries sector output (measured by landed value) could potentially generate 5 to 84 cents in household income output, and 28 cents to $3.34 in economic output. Nigeria appears to have the lowest income effect among the countries considered here, while Vietnam had the highest (Table 4). This suggests that an increase in fisheries landed value in Vietnam could potentially result in higher increases in household incomes relative to Nigeria, thereby providing Vietnamese fishery households with a better opportunity for improving their food security. Similarly, Vietnam also had the highest economic effect, while Sierra Leone had the lowest.

### Mitigating food security losses from high seas closure

#### Alternative sources of fish and non-fish food

High seas closure may adversely affect domestic fish supply in the 21 countries projected to experience net losses in catch under the 2 scenarios. On the positive side, it appears that all these countries had at least one other type of fishery that could potentially supplement the decreased catch of straddling fish taxa (Table 5). Inland and reef fisheries play an important role in providing subsistence catches for rural communities in Africa and the Pacific islands. Freshwater fish is also a crucial source of affordable protein for lower income groups in developing Asian countries [94]. The prevalence of inland and reef fisheries in the 21 countries indicates the importance of maintaining the sustainability of these fisheries resources and habitats in conjunction with marine coastal fisheries management, given that inland and reef fisheries are also overexploited where they occur [95–97].

**Table 5:** Presence of factors that may potentially dampen the effect of decreased fish supply due to high seas closure in countries projected to experience net catch losses.

. Country	Aquaculture	Inland/freshwater fisheries	Reef fisheries^§^ [131]	Food safety net programme†	% of agricultural land equipped for irrigation	Adaptive capacity	Fishing-farming/ livestock livelihoods	Global Food Security Index Score^≠^	References
Benin	√	√		2	0.35	Very low*	√	33.5	[40]
Comoros			√	1*	0.08	Very low*	√^	n/a^++^	[103,104]
Cote d’Ivoire	√^^	√		2	0.36	Very low	√	39.2	[105,106]
Fiji	√ ^	√	√	2*	0.7	Low	√	n/a^++^	[107,108]
Indonesia	√+	√	√	2	12.33	Low	√	47.7	[109,110]
Japan	√ ^^	√ ^		4	54.24	High	√^	77.9	[99]
Kiribati	√ ^		√	2*	0.31*	Low*	√^	n/a^++^	[55,111,112]
Korea	√ ^^+	√ ^		4	44.7	High	√^	72.1	[98]
Liberia	√+	√		1*	0.11	Very low*	√	n/a^++^	[58,113]
Malaysia	√ +	√^	√	2	4.64	Moderate	√	66.8	[114,115]
Mozambique	√	√^	√	1	0.24	Very low	√	31.0	[65,116]
Philippines	√^^	√	√	2	12.95	Moderate	√	49.8	[69]
Samoa	√^	√	√	2*	0.75*	Low*	√	n/a^++^	[70,108,112,117]
Seychelles	√^		√	1*	10	High**	√	n/a^++^	[118,119]
Sierra Leone	√	√		1	0.87	Very low	√	34.5	[120,121]
Sri Lanka	√	√	√	2	21.76	Low	√^	52.3	[79,122]
Tanzania	√	√	√	1	0.49	Very low	√	34.8	[110,123,124]
Thailand	√^+	√ ^^	√	3	30.46	Low**	√	58.7	[82,125,126]
Togo	√^	√		2	0.19	Very low	√	33.0	[127,128]
Vanuatu	√	√	√	2*	0.61*	Low	√	n/a^++^	[88,108,112]
Yemen	√^		√	1	2.9	Very low	√	36.1	[129,130]

^§^Reef fisheries are assumed to take place in all countries where coral reefs occur.

^†^ Based on a scale of 1 to 4 with 1 = Low presence, 4 = wide presence. See Methods for full explanation of rankings.

^≠^Source: Global Food Security Index. Countries with no scores available (n/a) are considered to be vulnerable to food insecurity based on for Pacific Island states, Comoros, and Liberia. Seychelles is considered to be a high income country that faces nutrition insecurity.

^^^ Limited.

^^^^ Important contributor to national fisheries production and/or for food security.

^+^ Emphasised for development to satisfy fish demand.

* No data/data deficient from cited source. Ranking is provided based on that of surrounding countries/country group.

** No data from [7]. Ranking is based on [126] for Thailand and [137] for Seychelles.

Japan and Korea, the two developed countries projected to experience catch losses, are likely the most capable of coping with decreased fish supply because their high wealth and trading power allows them to turn to international markets to obtain food. Further, although they have limited inland fisheries, both countries have high food security scores (above 70) and well developed aquaculture industries that are an important contributor to national production and food security [98,99]. With capture fisheries having levelled off globally, aquaculture is widely seen as the option to fill the future demand for fish [1,100], despite the debate over the environmental sustainability of certain aquaculture systems. However, while aquaculture presently plays a crucial role in providing an affordable source of protein for impoverished populations in developing countries of Asia and Africa [100], its expansion in low income food deficient countries may be limited by energy and technology demands [101]. It has also been argued that the nutritional quality of diets would drop if global fish supply becomes dominated by aquaculture [102].

Reef fisheries were assumed to take place in all countries where coral reefs occur [131] (Table 5). Places where inland and reef fisheries are most depended upon tend to be in tropical, developing countries where rapid population growth is occurring. In fact, the future availability of reef fish per capita in many Pacific island nations is expected to decrease due to population growth, and will be exacerbated by climate effects [15]. Consequently, we emphasise that the presence of alternative fish and/or food sources does not imply that there will be no problem when the projected decrease in straddling fish supply occurs. Population growth and other global change drivers may impede the alternative food sources from making up for the projected shortfall in straddling fish species supply.

Most of the 21 countries with projected losses (Table 5) are highly fish dependent and/or least developed countries, where the alternative food sources considered here are already being used to attain food security. In particular, African countries and island nations either had the lowest food security scores (less than 40) or were considered to be vulnerable to food insecurity [132–137] (Table 5), indicating that their food resources are already under stress, and it may not be possible to increase production in these food sectors. Non-fish alternatives already face substantial challenges–global crop production has to increase much more from current levels in order to meet the increased demand from population growth by 2050, and this is exacerbated by climate effects on rainfall and temperature [138]. In light of these considerations, it is important that the effect of global drivers on the potential for alternative food systems to make a substantial contribution to fish protein supply be taken into account in the context of high seas management.

Having a diversified livelihood portfolio is a way of increasing households’ resilience to shocks [139], and a means of reducing hunger and malnutrition for the rural poor [13]. Fishers in all negatively affected countries participated in diversified livelihoods by simultaneously engaging in fishing and farming, although farming opportunities for fishers was limited in the Comoros, Japan, and Korea. Nonetheless, the overall presence of diversified livelihoods in the affected countries is a positive sign that fishers may still be able to obtain food, albeit of different nutritional quality, in the event of decreased fish supply from high seas closure. Bushmeat is another alternate food source in times of low fish supply [140], but fish is still comparably cheaper than bushmeat, and thus preferred by the poor [41,141]. It is noted that the literature on fishers who participate in diversified livelihoods mainly referred to small-scale fishers; thus, the impact of decreased fish supply may be different for industrial fishers.

At the national level, the proportion of agricultural area that is equipped for irrigation can be used as an indicator for a country’s exposure to food supply shock [13]. In general, the availability of irrigated agricultural land in the African countries and Pacific islands considered here is very low. The prevalence of low irrigation reflects inadequate food production, and the projected decrease in fish supply may potentially exacerbate demands put on these countries’ already poor agricultural capacity. In particular, the loss of arable agricultural land in Pacific islands to housing and tourism development has already sparked concern [142]. The frequency of droughts and tropical cyclones in parts of Africa and the Pacific further impair food production [13,142]. In a global context, the irrigated area per person has been decreasing by 1% per year since 2000, and sources of irrigation water are scarce [143]. Both these factors may contribute to decreased opportunities for securing alternate sources of food in countries projected to experience net losses in domestically consumed fish due to high seas closure.

The countries with lowest adaptive capacity levels were also concentrated in Africa, where poor infrastructure, low human capital in many coastal areas, and political stability are among the factors which channel through food systems and hinder people from obtaining a stable supply of food [144]. This highlights that improving food security also encompasses overcoming the social, economic, and institutional constraints for coping with external stressors [145] such as climate change (e.g., drought), conflicts, and disease. In terms of the factors investigated here, it appears that Japan and Korea have the best potential for mitigating the effects of decreased domestic fish supply due to high seas closure, whereas Comoros and Yemen have the least opportunity for doing so.

#### Inequality and governance

Inequitable distribution of resources and poor governance institutions can create barriers for sustainable food systems and societal well-being, thereby ultimately affecting food security. In Comoros, the lack of alternate food sources is exacerbated by high income inequality and poor governance effectiveness, while in Yemen opportunities for food security may be hampered by high levels of corruption, and poor political stability and governance effectiveness, relative to all other assessed countries (Table 6). In contrast, the economic and governance conditions in Japan puts it in a much better position for achieving food security, as it has the highest levels of income equality and sound governance among the countries. Compared across countries, income inequality may pose the biggest barrier in Seychelles. Thailand, Cote d’Ivoire, and the Philippines had the highest political instability, which can potentially restrict the availability and access to food (see the example of Cote d’Ivoire in [146]). Poor governance effectiveness may also hamper food security measures in 3 other African countries—Liberia, Sierra Leone, and Togo. This amplifies the already poor prospects for alternate non-fish sources of food in these countries, given that they also have limited agriculture and food safety net programmes (Table 5). In contrast, while Pacific island nations also have limited agricultural potential, they generally have more favourable governance conditions and income equality relative to African countries.

**Table 6:** Summary of Gini coefficient and governance indicators for countries projected to experience net catch losses.

Country	Gini index^1^	Control of corruption^2^	Governance effectiveness^2^	Political stability^2^
Benin	38.6	-0.78	-0.50	0.05
Comoros	64.3	-0.53	-1.67	-0.19
Cote d’Ivoire	41.5	-0.41	-0.78	-1.01
Fiji	42.8	-0.03	-0.37	0.48
Indonesia	38.1	-0.58	-0.01	-0.37
Japan	32.1	1.73	1.82	1.02
Kiribati	37.6	0.31	-0.58	0.72
Korea	31.3	0.49	1.18	0.19
Liberia	38.2	-0.78	-1.37	-0.63
Malaysia	46.2	0.48	1.14	0.34
Mozambique	45.7	-0.70	-0.73	-0.35
Philippines	43.0	-0.44	0.19	-0.70
Samoa	42.7	0.32	0.43	1.15
Seychelles	65.8	0.37	0.39	0.42
Sierra Leone	35.4	-0.95	-1.22	-0.22
Sri Lanka	36.4	-0.34	0.09	-0.25
Tanzania	37.6	-0.80	-0.64	-0.54
Thailand	39.4	-0.41	0.34	-0.91
Togo	39.3	-0.92	-1.26	-0.16
Vanuatu	37.2	0.62	-0.55	0.66
Yemen	37.7	-1.55	-1.41	-2.53

^1^A Gini score of 0 represents perfect equality and 1 represents perfect inequality. Note that we converted the Gini index provided by [22], which initially ranged from 0–100 to a range of 0–1.

^2^Scores range from approximately -2.5 to +2.5, with higher values corresponding to better governance.

We acknowledge that this qualitative assessment deals primarily with availability of fish, and does not fully consider the other three dimensions of food security (accessibility, affordability, and utilization). Future research can therefore incorporate other vital factors that determine who will ultimately benefit from improved food security, e.g., people’s access to livelihoods in fish value chains and the affordability of fish [1].

Our results are built on the projected impacts of high seas closure on individual countries’ catches, which may be affected by the underlying model assumptions from [11]. Briefly, these assumptions included: 1) the catch data used were representative of true fisheries catches (i.e., insignificant misreporting of straddling taxa); 2) increased catches of straddling taxa was applied evenly across all EEZs without accounting for geographic and interspecific variation arising from the accuracy of reported data and the potential spillover of biomass from closed high seas areas. Both these assumptions could possibly affect the magnitude of projected changes in catch. For instance, IUU (illegal, unreported, and unregulated) fishing not captured in catch statistics could result in lower than expected gains of straddling fish taxa to certain countries. Importantly, we stress that the projected benefits arising from high seas closure can only be realised if fisheries within each EEZ are themselves managed well. The outcomes presented here are also subject to climate effects on the spatial and biological behaviour of straddling fish taxa, which were not accounted for in the underlying model. However, recent research suggests that closing the high seas to fishing or managing its fisheries cooperatively could increase catches in EEZs by around 10% by 2050 under 2 climate change scenarios [12].

## Summary and Concluding Remarks

The purpose of this study was to investigate the effect high seas closure would have on the availability of commonly consumed food fish in fish reliant, low income countries. We find that just above half (54%) of the assessed countries used straddling fish taxa locally, and hence would potentially be affected by high seas closure. At the same time, countries which did not consume straddling fish taxa domestically could also be indirectly affected, for instance, through the loss in fishing access fees. Overall, it appears that high seas closure affected more countries positively than negatively in terms of improving catches of straddling fish taxa; however, the magnitude of projected losses for negatively affected countries exceeded projected gains. Moreover, only slightly more than a third (37%) of the countries where straddling fish taxa were consumed domestically were projected to experience an increase in fish supply under both scenarios. It should be noted that since future consumption levels of straddling stocks is likely to change in these countries, this conclusion could change in the near future.

The majority (64%) of both highly fish dependent countries (HFDCs) and least developed countries (LDCs) made use of straddling taxa domestically. Slightly above half (57%) the HFDCs were projected to gain in terms of increased fish supply, while almost 30% would be negatively affected across both scenarios. Among LDCs, 20% of the countries were projected to be negatively affected across both scenarios. Least developed countries that are highly dependent on fish (HFDLDCs) are arguably the countries with the greatest need for improved access to affordable fish for food and nutrition security. However, among all country groups, HFDLDCs had the lowest proportion (33%) that used straddling fish taxa domestically. Across both scenarios, slightly above a quarter (28%) of HFDLDCs would likely benefit from high seas closure in terms of increased fish availability, and only one of the countries was projected to be negatively affected.

Thus, in general, high seas closure may not have a substantial impact on improving fish supply in countries where it is most needed. At the same time, local food security for HFDLDCs that were projected to fare the worst (i.e., losses in both catch scenarios) may not be heavily impacted because the affected straddling taxa are tuna, which are either used for export or are caught by foreign fishing fleets. Nevertheless, high seas closure may affect food security indirectly through economic effects stemming from loss in exports and foreign fishing access fees. In summary, while high seas closure may benefit local fish supply in less than half the assessed countries overall, it is important to bear in mind that countries projected to experience catch gains but where straddling taxa are not used domestically can still attain food security benefits indirectly through economic and household income effects arising from an increase in fisheries output.

Protecting the high seas is a conservation issue that concerns the global community. Although prior studies have shown that high seas protection is likely to provide ecological benefits, this study is, as far as we are aware, the first to investigate the food security impact of high seas closure on the world’s poorest and most fish dependent countries. Our results indicate that while it may not likely improve domestic fish supply substantially in these countries, its negative impact upon food security in these countries also appears to be minimal. Furthermore, fish catch increases arising from high seas closure can indirectly contribute to improved food security via other economic activities in countries where straddling fish taxa are not consumed domestically. At the same time, this also implies that indirect negative impacts may be experienced in those countries which do not consume straddling fish taxa domestically. In particular, a decrease in tuna catches may not only result in certain Pacific Island States losing substantial amounts of fishing access fees, but also a source of future food security in the face of climate change.

Although it is beyond the scope of this study to consider the political and technological requirements of high seas protection, our results suggest that high seas closure can potentially benefit biodiversity loss and food insecurity, which were identified by the Millennium Assessment as two of the biggest challenges facing humanity. However, we also caution that high seas closure can negatively impact food security in some countries, and that this impact will be particularly amplified in those that are already highly fish dependent and low income. By doing so, our study provides a starting point for further evaluation of the costs and benefits of high seas protection, an international action that is urgently needed in the face of global ocean degradation.

## Supporting Information

S1 Table Average projected % change in catch relative to the status quo for countries which use and do not use straddling taxa domestically, according to country groups (Least Developed Countries (LDCs), High Fish Dependent Countries (HFDCs), and High Fish Dependent LDCs (HFDLDCs)), under 5 scenarios of catch gains following high seas closure. Data source: Sumaila et al. (2015).
(DOCX)

S2 Table Projected change in catch and landed value for Least Developed Countries (LDCs) under 5 scenarios of straddling taxa catch increase following high seas closure. LDCs are listed in order of their dependence on fish as a source of animal protein. Source: Sumaila et al. (2015).
(DOCX)

S3 Table High Fish Dependent Countries (HFDCs) and projected percentage change in catch and landed value under 5 scenarios of straddling taxa catch increase following high seas closure Source: Sumaila et al. (2015). (DOCX)
