# Supplementary material for: Impact of High Seas Closure on Food Security in Low Income Fish Dependent Countries
Source: PLoS One. 2016 Dec 29;11(12):e0168529. doi: 10.1371/journal.pone.0168529 (PMC5199032; doi:10.1371/journal.pone.0168529)
Supplement: S1 Table — Data source: Sumaila et al. (2015). (DOCX) [file pone.0168529.s001.docx]

|  | Scenario | | | | |
| --- | --- | --- | --- | --- | --- |
|  | 10% | 18% | 20% | 42% | 70% |
| Domestic use |  |  |  |  |  |
| HFDC | -12.0 | -7.3 | -5.9 | 8.0 | 25.6 |
| HFDLDC | -9.6 | -5.8 | -4.7 | 6.6 | 20.8 |
| LDC | -4.8 | -0.3 | 1.1 | 14.2 | 30.8 |
|  |  |  |  |  |  |
| No domestic use |  |  |  |  |  |
| HFDC | -30.2 | -25.7 | -24.4 | -11.1 | 5.7 |
| HFDLDC | -7.5 | -1.5 | 0.3 | 18.0 | 40.2 |
| LDC | -10.8 | -7.1 | -3.4 | 13.5 | 35.9 |
