# Supplementary material for: Impact of High Seas Closure on Food Security in Low Income Fish Dependent Countries
Source: PLoS One. 2016 Dec 29;11(12):e0168529. doi: 10.1371/journal.pone.0168529 (PMC5199032; doi:10.1371/journal.pone.0168529)
Supplement: S2 Table — LDCs are listed in order of their dependence on fish as a source of animal protein. Source: Sumaila et al. (2015). (DOCX) [file pone.0168529.s002.docx]

| **COUNTRY** | **CATCH** | | | | | **LANDED VALUE** | | | | |
| --- | --- | --- | --- | --- | --- | --- | --- | --- | --- | --- |
|  | **10%** | **18%** | **20%** | **42%** | **70%** | **10%** | **18%** | **20%** | **42%** | **70%** |
| Solomon Is. | 12.1 | 21.3 | 24.0 | 51.0 | 84.9 | 12.1 | 21.3 | 24.0 | 51.0 | 84.9 |
| Kiribati | -45.6 | -42.1 | -41.0 | -30.7 | -17.6 | -45.6 | -38.4 | -37.5 | -28.0 | -16.0 |
| Sierra Leone | -0.8 | 1.4 | 2.1 | 8.6 | 16.8 | -0.8 | 1.6 | 2.4 | 9.9 | 19.5 |
| Tuvalu | 12.1 | 21.3 | 24.0 | 51.0 | 84.9 | 12.1 | 21.3 | 24.0 | 51.0 | 84.9 |
| Cambodia | 12.1 | 21.3 | 24.0 | 51.0 | 84.9 | 12.1 | 21.3 | 24.0 | 51.0 | 84.9 |
| Eq. Guinea | 12.1 | 21.3 | 24.0 | 51.0 | 84.9 | 12.1 | 21.3 | 24.0 | 51.0 | 84.9 |
| Comoros | -83.2 | -82.3 | -82.0 | -79.4 | -76.0 | -83.2 | -81.1 | -80.8 | -78.2 | -74.9 |
| Bangladesh | 2.4 | 4.3 | 4.8 | 10.2 | 17.0 | 2.4 | 18.4 | 20.8 | 44.0 | 73.4 |
| Vanuatu | -86.2 | -85.0 | -84.7 | -81.4 | -77.2 | -86.2 | -85.0 | -84.7 | -81.4 | -77.2 |
| Gambia | 1.7 | 4.4 | 5.2 | 13.1 | 23.1 | 1.7 | 6.7 | 7.9 | 19.9 | 35.0 |
| Sao Tome Principe | 11.4 | 20.1 | 22.6 | 47.9 | 79.8 | 11.4 | 19.7 | 22.2 | 47.1 | 78.5 |
| Senegal | 4.2 | 8.5 | 9.7 | 22.4 | 38.3 | 4.2 | 6.3 | 7.3 | 16.7 | 28.6 |
| Congo Dem Rep | 7.1 | 15.0 | 17.3 | 40.4 | 69.6 | 7.1 | 15.2 | 17.6 | 41.0 | 70.6 |
| Togo | -12.0 | -9.5 | -8.8 | -1.5 | 7.8 | -12.0 | -7.6 | -7.0 | -1.2 | 6.2 |
| Myanmar | 12.1 | 21.3 | 24.0 | 51.0 | 84.9 | 12.1 | 21.3 | 24.0 | 51.0 | 84.9 |
| Mozambique | -1.8 | 5.3 | 7.4 | 28.2 | 54.4 | -1.8 | 5.2 | 7.3 | 27.7 | 53.5 |
| Benin | -8.4 | -4.9 | -3.8 | 6.7 | 19.9 | -8.4 | -4.9 | -3.8 | 6.8 | 20.1 |
| Guinea | 1.0 | 1.7 | 2.0 | 4.2 | 7.0 | 1.0 | 1.3 | 1.5 | 3.2 | 5.4 |
| Angola | 6.6 | 11.9 | 13.5 | 29.1 | 48.7 | 6.6 | 8.6 | 9.7 | 21.0 | 35.2 |
| Samoa | -91.6 | -91.2 | -91.0 | -89.7 | -88.0 | -91.6 | -89.3 | -89.2 | -87.9 | -86.2 |
| Tanzania | -33.9 | -32.3 | -31.8 | -26.9 | -20.7 | -33.9 | -27.9 | -27.4 | -23.0 | -17.9 |
| Madagascar | 6.1 | 10.7 | 12.0 | 25.5 | 42.5 | 6.1 | 10.6 | 11.9 | 25.3 | 42.1 |
| Haiti | 12.1 | 21.3 | 24.0 | 51.0 | 84.9 | 12.1 | 21.3 | 24.0 | 51.0 | 84.9 |
| Mauritania | 9.3 | 16.6 | 18.7 | 39.7 | 66.3 | 9.3 | 13.9 | 15.7 | 33.4 | 55.7 |
| Timor Leste | 9.9 | 17.5 | 19.7 | 41.7 | 69.5 | 9.9 | 9.6 | 10.9 | 23.0 | 38.4 |
| Yemen | -41.9 | -39.1 | -38.3 | -30.2 | -19.9 | -41.9 | -39.6 | -38.7 | -30.5 | -20.1 |
| Liberia | -6.6 | -1.0 | 0.6 | 16.8 | 37.3 | -6.6 | -0.9 | 0.6 | 15.5 | 34.3 |
| Djibouti | 6.8 | 11.9 | 13.5 | 28.5 | 47.5 | 6.8 | 10.0 | 11.3 | 23.9 | 39.8 |
| Guinea Bissau | 6.9 | 1.7 | 13.7 | 29.2 | 48.6 | 6.9 | 1.4 | 12.6 | 26.8 | 44.7 |
| Somalia | 6.6 | 11.6 | 13.1 | 27.8 | 46.3 | 6.6 | 3.2 | 3.6 | 7.6 | 12.7 |
| Eritrea | 4.1 | 7.3 | 8.2 | 17.3 | 28.9 | 5.20 | 9.1 | 10.3 | 21.8 | 36.4 |
| Sudan | 8.5 | 14.9 | 16.8 | 35.6 | 59.4 | 5.31 | 9.3 | 10.5 | 22.3 | 37.2 |
| **Average** | **-7.7** | **-3.0** | **-1.1** | **13.7** | **32.5** | **-7.6** | **-3.0** | **-1.3** | **12.9** | **30.8** |
