# Supplementary material for: Impact of High Seas Closure on Food Security in Low Income Fish Dependent Countries
Source: PLoS One. 2016 Dec 29;11(12):e0168529. doi: 10.1371/journal.pone.0168529 (PMC5199032; doi:10.1371/journal.pone.0168529)
Supplement: S3 Table — (DOCX) [file pone.0168529.s003.docx]

| **COUNTRY** | **CATCH** | | | | | **LANDED VALUE** | | | | |
| --- | --- | --- | --- | --- | --- | --- | --- | --- | --- | --- |
|  | 10% | 18% | 20% | 42% | 70% | 10% | 18% | 20% | 42% | 70% |
| Maldives | 12.1 | 21.3 | 24.0 | 51.0 | 84.9 | 12.1 | 21.3 | 24.0 | 51.0 | 84.9 |
| Indonesia | -4.0 | 0.1 | 1.3 | 13.1 | 28.0 | -3.5 | 0.1 | 1.1 | 11.6 | 24.9 |
| Seychelles | -83.7 | -82.5 | -82.1 | -78.3 | -73.6 | -83.7 | -82.4 | -82.1 | -78.3 | -73.6 |
| Sri Lanka | -53.6 | -49.9 | -48.8 | -37.9 | -24.2 | -52.8 | -49.1 | -48.1 | -37.4 | -23.9 |
| Japan | -12.0 | -6.7 | -5.2 | 10.2 | 29.7 | -13.5 | -7.6 | -5.8 | 11.5 | 33.4 |
| Philippines | -7.2 | -1.4 | 0.3 | 17.4 | 38.9 | -6.5 | -1.2 | 0.3 | 15.5 | 34.8 |
| Korea Rep | -33.3 | -29.5 | -28.3 | -17.1 | -2.8 | -33.8 | -29.9 | -28.7 | -17.3 | -2.9 |
| Thailand | -4.2 | 1.1 | 2.6 | 18.1 | 37.6 | -3.8 | 1.0 | 2.3 | 16.2 | 33.6 |
| Malaysia | -15.5 | -11.8 | -10.7 | 0.2 | 13.9 | -18.0 | -13.6 | -12.4 | 0.2 | 16.1 |
| Cameroon | 1.6 | 3.1 | 3.6 | 8.2 | 13.9 | 1.5 | 3.0 | 3.4 | 7.9 | 13.4 |
| Cote d'Ivoire | -4.2 | 0.9 | 2.4 | 17.4 | 36.3 | -3.9 | 0.9 | 2.3 | 16.3 | 34.0 |
| Nigeria | 7.1 | 12.7 | 14.3 | 30.7 | 51.4 | 5.6 | 10.1 | 11.4 | 24.3 | 40.7 |
| Vietnam | 12.1 | 21.3 | 24.0 | 51.0 | 84.9 | 12.1 | 21.3 | 24.0 | 51.0 | 84.9 |
| Fiji | -56.4 | -54.4 | -53.8 | -47.7 | -40.1 | -50.3 | -48.4 | -47.9 | -42.5 | -35.7 |
| **Average** | **-17.2** | **-12.5** | **-11.2** | **2.6** | **19.9** | **-17.0** | **-12.5** | **-11.1** | **2.1** | **18.9** |
